# NFBLab—A Versatile Software for Neurofeedback and Brain-Computer Interface Research

**DOI:** 10.3389/fninf.2018.00100

**Published:** 2018-12-24

**Authors:** Nikolai Smetanin, Ksenia Volkova, Stanislav Zabodaev, Mikhail A. Lebedev, Alexei Ossadtchi

**Affiliations:** ^1^Center for Bioelectric Interfaces, National Research University Higher School of Economics, Moscow, Russia; ^2^Medical Computer Systems, Zelenograd, Russia

**Keywords:** neurofeedback, low-latency, software, real-time EEG, brain-computer interface, flexible experiment design, LSL-protocol

## Abstract

Neurofeedback (NFB) is a real-time paradigm, where subjects learn to volitionally modulate their own brain activity recorded with electroencephalographic (EEG), magnetoencephalographic (MEG) or other functional brain imaging techniques and presented to them via one of sensory modalities: visual, auditory or tactile. NFB has been proposed as an approach to treat neurological conditions and augment brain functions. Although the early NFB studies date back nearly six decades ago, there is still much debate regarding the efficiency of this approach and the ways it should be implemented. Partly, the existing controversy is due to suboptimal conditions under which the NFB training is undertaken. Therefore, new experimental tools attempting to provide optimal or close to optimal training conditions are needed to further exploration of NFB paradigms and comparison of their effects across subjects and training days. To this end, we have developed open-source NFBLab, a versatile, Python-based software for conducting NFB experiments with completely reproducible paradigms and low-latency feedback presentation. Complex experimental protocols can be configured using the GUI and saved in NFBLab's internal XML-based language that describes signal processing tracts, experimental blocks and sequences including randomization of experimental blocks. NFBLab implements interactive modules that enable individualized EEG/MEG signal processing tracts specification using spatial and temporal filters for feature selection and artifacts removal. NFBLab supports direct interfacing to MNE-Python software to facilitate source-space NFB based on individual head models and properly tailored individual inverse solvers. In addition to the standard algorithms for extraction of brain rhythms dynamics from EEG and MEG data, NFBLab implements several novel in-house signal processing algorithms that afford significant reduction in latency of feedback presentation and may potentially improve training effects. The software also supports several standard BCI paradigms. To interface with external data acquisition devices NFBLab employs Lab Streaming Layer protocol supported by the majority of EEG vendors. MEG devices are interfaced through the Fieldtrip buffer.

## 1. Introduction

In the biological feedback (BF) paradigm, subjects gain the ability to monitor and control the physiological parameters of their own bodies, recorded with artificial sensors. In the case of neurofeedback (NFB), subjects learn to control the activity of their brains (Kamiya, 1969; Sterman et al., 1974; Sitaram et al., 2016; Ossadtchi et al., 2017). In NFB settings, neural activity is recorded with such methods as electroencephalography (EEG), magnetoencephalography (MEG), electrocorticography (ECoG), functional magnetic resonance imaging (fMRI) or multielectrode implants and converted into stimuli that subjects can perceive. NFB processing stages include recording of neural activity, extracting features of interest from the recorded signals, feature transformation, and delivering the feedback signal to the subject via one of the sensory modalities, most often visual, auditory or tactile. A very similar processing scheme is utilized in brain-computer interfaces (BCIs), the systems that connect the brain to external assistive devices (Wolpaw, 2012). In BCIs, the feedback is implemented in the form of response of the external device to the commands derived from the neural activity of the user; this signal can be considered as a type of NFB.

NFB and BCI paradigms are utilized in neurophysiological research, clinical practice, and consumer applications, like video games, meditation and performance training gadgets. In the clinical world, NFB methods often complement conventional approaches to treatment of neurological disorders (Coben et al., 2010; Lofthouse et al., 2012) and cognitive enhancement training (Zoefel et al., 2011). EEG-based BCIs can be used for post-stroke neurorehabilitation (Ang et al., 2015; Frolov et al., 2018), where motor intentions are extracted from brain activity and directed to exoskeleton devices or functional electrical stimulators that evoke limb movements, which in turn generate sensory re-afferent signals that flow back to the brain and mediate restorative brain plasticity.

EEG-based NFB systems have become widespread because EEG recordings are noninvasive, relatively simple to implement, and can be informative of many neural functions. In modern implementations, multichannel EEG signals are amplified, digitized, and transmitted to a computer for denoising and feature extraction. EEG features are often derived from oscillatory components of the multichannel EEG data, such as the theta (3–7 Hz), alpha (8–12 Hz), mu (9–14 Hz), and beta (15–25 Hz) rhythms. The neural mechanisms underlying different EEG features depend on the rhythm frequency and cortical source. NFB therapy usually attempts to correct a targeted physiological function by attempting to increase or decrease particular EEG rhythm power. In clinical settings, quantitative EEG (QEEG) technology is utilized to detect the difference between the EEG of a patient and database values for healthy controls. Many QEEG methods incorporate statistical analyses of the EEG spectral bands. Additionally, the group independent component analysis (gICA) has been developed, where EEG dynamics of a patient is compared to a database of independent components (Ponomarev et al., 2012).

The efficiency of NFB training critically depends on the appropriate selection of the spatial, spectral and temporal characteristics of the feedback signal. To ensure that these characteristics reflect in a timely fashion the neural functions of interest, several spatial and temporal filtering methods have been proposed that isolate signal of interest from noise and concurrent brain activity present in multichannel EEG/ECoG/MEG data, with minimal possible latency. Spatial filtering is implemented based on the signal analysis by means of popular algorithms such as principal component analysis (PCA), common spatial pattern (CSP) (Koles et al., 1990), independent component analysis (ICA) (Bell and Sejnowski, 1995), and spatio-spectral decomposition (SSD) (Nikulin et al., 2011) etc. Temporal filtering is then applied to the extracted spatial component in order to extract the signal from specific frequency band and estimate its instantaneous power (Lee et al., 2006; Smetanin and Ossadtchi, 2017). Individual variability of EEG rhythms is an important factor that affects NFB efficiency. Ideally, each subject's anatomical and physiological features should be taken into consideration and NFB-generating algorithms adjusted accordingly. Such adjustment is, however, rarely done, which causes sub-optimal performance of NFB. Overall, there is still no established procedure that ensures a reliable and efficient NFB. Unimpressive performance of several NFB implementations, particularly the ones with double blind controls, has caused criticism toward the NFB paradigm as a whole (Sitaram et al., 2016).

Among the obstacles hindering the achievement of better results with NFB, are the limitations of the existing software tools, which in many cases are cumbersome to use, are not flexible and do not enable certain desirable features. To address this range of problems, here we report a new software, the NFBLab, suitable for conducting many NFB and BCI experiments. The platform incorporates both standard and novel algorithms for real-time processing of EEG/MEG/ECoG activity and generating NFB. The key features of this software include:

- Support of the most common EEG/MEG devices using the lab streaming layer (LSL)^1^ and FieldTrip buffer^2^;
- Internal language that allows implementing different experimental protocols using XML-formatted descriptions;
- Graphical user interface for design of signal processing tracts and to form flexible sequences of experimental blocks;
- Interactive module for configuring signal processing pipelines based on the test data collected in individual subjects;
- Support of EEG/MEG inverse problem solvers (e.g., wMNE and LCMV beamformers) using direct interaction with MNE-Python software;
- Continuous visualization of signal features;
- Support of mock feedback and experimental blocks randomization;
- New in-house algorithms for low-latency processing of narrow-band signals (Smetanin and Ossadtchi, 2017);
- Open-source Python code^3^ and cross-platform compatibility.

## 2. Comparable Software

Several software platforms are currently available that furnish flexibility needed for conducting experiments that involve real-time processing and visualization of multichannel bioelectric signals. Among them, OpenVIBE (Renard et al., 2010) is a very popular, actively supported project. It incorporates visual-programming tools and scripts for configuring complex signal processing pipelines. An experimental task is usually subdivided into several parts specified in separate XML files (e.g., a motor-imagery BCI^4^). Each part is launched manually, which may adversely affect reproducibility of the conducted experiments. Additionally, this software is not open-source.

BCI2000 is another popular software solution for implementing NFB and BCI paradigms (Schalk et al., 2004). This is a closed-source C/C++ software with several built-in algorithms for processing neural activity and extracting signals of interest. Like OpenVIBE, different experimental tasks are launched manually in BCI2000. BCI2000 supports integration with external programs and MATLAB environment, which allows users to incorporate additional data processing modules.

The NFBLab software proposed here advances the functionality of both OpenVIBE and BCI2000 platforms. One innovation is the NFBLab's capacity to configure signal processing pipelines based on the data collected in individual subjects. This functionality incorporates a rich set of decompositions (PCA, ICA, CSP, SSD) for extracting essential neural modulations while discarding noise. Furthermore, the NFBLab software contains an advanced experiment control module that enables tasks consisting of multiple blocks. The blocks can be automatically launched, switched, repeated and randomized. Mock NFB is supported, as well. NFBLab is written in Python. The code is open-source, which facilitates sharing and improving these software tools by the developers of NFB and BCI systems. The code and installation instructions can be found at https://github.com/nikolaims/nfb repository.

In addition to Open VIBE, BCI2000 and our NFBLab platform, several commercial clinical software platforms should be mentioned that have been designed for NFB therapy. These are BrainMaster, NeuroRT Training, Cygnet and several others. These systems should be clearly delineated from the research oriented software mentioned earlier. They operate under a very harsh requirement for exclusively high usability within the clinical environment which often adversely affects their flexibility and the fidelity of training protocols. While some of these platforms support spatial filtering of neural signals, the parameters of such filters are obtained from solving the generic inverse problem without taking into account the anatomical and functional parameters of individual subjects. Thus, sLORETA (Pascual-Marqui, 2002) with a generic head model is commonly utilized within popular NFB software. With this approach, the output signal could be different from the individual subject's true activity of the brain region of interest (ROI). Additional inaccuracies are introduced to the performance of forward models by the unknown tissue conductivity profiles. Furthermore, NFB systems suffer from the presence of delay between neural modulations and NFB delivery. This delay is typically more than 500 ms, which could be too long for NFB to be efficient (Larsen and Sherlin, 2013).

NFBLab offers solutions to both spatial and temporal NFB specificity problems. In the spatial domain, there is an option to use a subject-specific spatial filter. The filter parameters are derived based on the spatial-temporal decomposition (PCA, ICA, CSP, SSD) applied to the EEG/MEG/ECoG data recorded during functional tests, for example opening/closing the eyes, blinking, and performing hand movements. These functional tests allow for building subject-specific spatial filters without the need to perform laborious calculations, such as constructing forward models from the individual MRI data. Additionally, NFBLab supports conventional ROI-based approaches by importing, via the MNE-Python package, inverse operators calculated using a broad range of methods (Darvas et al., 2004) based on the generic or individual anatomy.

In the temporal domain, NFBLab incorporates several advanced signal processing algorithms that effectively decrease NFB latency. These algorithms process EEG signals and extract envelopes of their narrow-band components with minimal possible or even negative latency.

## 3. Architecture

**Figure 1:**
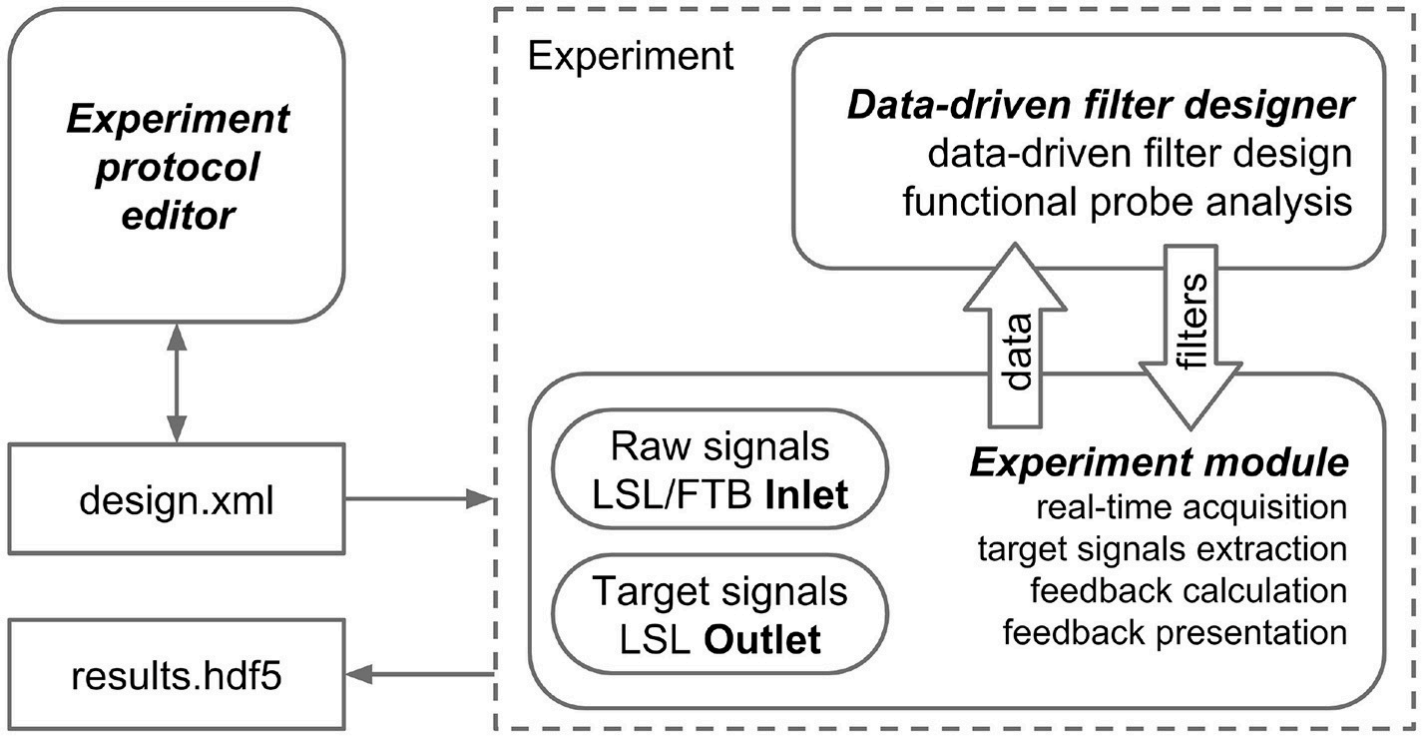
NFBLab architecture. Three main modules comprise NFBLab. *Experiment protocol editor* details experimental task. It describes the sequence of experimental blocks, properties of the individual blocks, sequence of blocks within the experiment, signal processing procedures used NFB presentation parameters. *Data-driven filter designer* allows to interactively edit signal processing flow and construct spatial and temporal filters. The filters are based on the frequency analysis and spatial decomposition of the EEG data obtained from the functional tests. *Experiment module* does actual real-time processing and displays the raw and transformed neural signals. It also controls the sequence of experimental blocks and the presentation of stimuli and NFB.

The NFBLab platform consists of three main modules, as illustrated in Figure 1. The first module, called “Experiment protocol editor,” specifies an experimental task. It describes the sequence of experimental blocks, signal processing procedures to compute target signals and the parameters used to calculate NFB within each experimental block. The block descriptions are saved as XML files, which are then loaded into the second module, called “Experiment module,” which needs to be launched at the start of the experiment. This XML-language based structure allows for reproducibility of experiments. Experimental module processes and displays the raw and transformed neural signals. The Experiment module also controls the sequence of experimental blocks and the presentation of stimuli and NFB. The third module, Data-driven filter designer, is an interactive module for editing signal processing flow and constructing spatial and temporal filters. The filters are based on the frequency analysis and spatial decomposition of the EEG data obtained from the functional tests. Short latency data exchange with EEG/MEG/ECoG recording devices is mediated by the LSL or FieldTrip buffer technologies. The experimental data along with the synchronized feedback signal is saved as HDF5 files, and output signals are streamed to the LSL outlet for communication with the external programs and devices. The relationships between NFBLab modules is shown in Figure 1.

### 3.1. Support of External Devices

The main instrument for working with external devices in NFBLab is the Lab Streaming Layer (LSL) protocol. LSL is a socket based data transfer protocol that controls synchronized collection and transmission of multi-channel time series. The protocol can also add meta-information, for example, names of devices and recording channels. The central element of LSL is a multichannel data stream whose input/output operations are supported by *LSL Inlet* and *LSL Outlet* objects, correspondingly.

Data streaming from the EEG/MEG/ECoG devices to the NFBLab is carried out through the LSL protocol typically implemented within a separate independent module that reads data following the specifications of the specific acquisition device and converts it into a standard LSL stream. Acquisition software of some manufacturers of EEG recording devices includes LSL streaming feature, for example NeoRec software (Medical Computer Systems, Ltd.) and EEG Studio (Mitsar Co., Ltd). NFBlab intercepts such streams by name and allows selecting/excluding channels, and defining a new EEG reference.

NFBLab supports multiple LSL streams running simultaneously, for example simultaneous data acquisition and processing with several external devices. These are typically EEG/ECoG/MEG recording systems, but other types of data can be streamed, as well, such as multichannel electromyography, thermometry, eye-tracking and limb kinematics acquired using motion capture systems.

The NFBLab interface allows to set up a broadcast of input and output signals, and their visualization. For test purposes, previously recorded data could be replayed as an LSL stream at a specified sampling rate, which allows designing experimental tasks without the need to connect to any external equipment. In addition to the LSL streams, NFBLab supports FieldTrip buffer, an alternative data transfer protocol, which allows for nearly real-time communication with magnetoencephalographic acquisition devices.

## 4. Signal Processing Pipeline Specification

**Figure 2:**
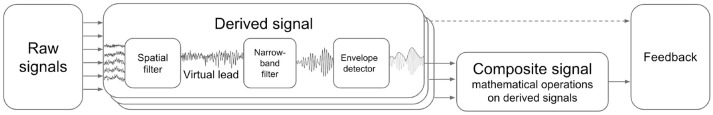
Derived and composite signals processing pipeline. Virtual lead is obtained by means of spatial filtering operation applied to EEG channels, this corresponds to computing a linear combination of EEG channel signals with coefficients aimed to emphasize features of interest and suppress the interference. Derived signal usually represents envelope of the narrow-band filtered virtual lead signal. Composite signal can be computed by applying an arbitrary user-specified mathematical function to a pair of derived signals. Coherence represents a special case when the composite signal is obtained based on the pair of virtual signals (not derived signals).

NFBLab implements *virtual derivations* (or virtual leads) and applies narrow-band filtering followed by envelope extraction to obtain *derived signals* representing instantaneous power within specific EEG band. It also allows for mathematical operations on pairs of *derived signals* to obtain *composite signals*. Appropriately scaled *derived* and *composite signals* can be then presented to a subject as NFB. Figure 2 schematically illustrates this processing structure.

### 4.1. Virtual Derivations and Derived Signals

Here and below, virtual derivation (virtual lead) is defined as an instantaneous linear combination of the signals from different channels stored in vector **x**(*t*), which formally can be written as *y*(*t*) = **w**^*T*^**x**(*t*). Although it is not exactly technically correct, in the EEG signal processing community this operation is referred to as spatial filtering. The weight vector **w** of this linear combination can be trivial, that is it can consist of all zeros except for “1” in a single position. In this case, the virtual derivation is simply the signal from the channel corresponding to the index of the non-zero element of the weight vector.

In the source-space NFB paradigm (Congedo et al., 2004), NFB represents activity of a cortical ROI. This signal is usually calculated by solving the EEG/MEG inverse problem, where an *M*×*N* inverse operator matrix **H** = {*h*_*ij*_}, (*i, j*) ∈ [(1, *M*), (1, *N*)] is calculated with the *i*−th row ${\text{h}}_{i}^{T}$ corresponding to the *i*−th ROI. The ROI activity **y**_*i*_(*t*) is derived from the *N*-channel signal **x**(*t*) as a dot product ${\text{y}}_{i}\left(t\right)={\text{h}}_{i}^{T}\text{x}\left(t\right)$. Thus, the spatial filter vector ${\text{w}}^{T}={\text{h}}_{i}^{T}$. While there exist a plethora of methods for solving the EEG/MEG inverse problem (Darvas et al., 2004), most of the implementations of source space neurofeedback rely on the generic sLoreta (Pascual-Marqui, 2002) solver based on the standardized head model. NFBLab implements sLoreta-based neurofeedback, as well, but also provides an interface to a full-blown inverse problem solver implemented in MNE-Python software, including several versions of the Minimum Norm Estimates (Hamalainen and Ilmoniemi, 1994) and beamforming approaches (Greenblatt et al., 2005).

The accuracy of the inverse modeling approaches suffers from the individual variability in head-shapes and spatial profiles of head tissue conductivity. The conductivity effects are especially strong when EEG-based NFB is used because tissue conductivity significantly affects the electrical potentials recorded from the scalp. To circumvent this problem, NFBLab offers an alternative approach for building spatial filters. This approach is based on the use of functional tests. One version of this approach was previously reported (White et al., 2014). In a general case, this approach consists of collecting segments of EEG/ECoG/MEG data during functional tasks, such as eyes opening/closing, rotating the hand, blinking, relaxing and several others. These tasks yield EEG/ECoG/MEG data that can be used for identifying patterns of interest, which are localized in space-time-frequency domain and reflect functionally relevant modulations of the underlying neuronal activity. Meaningful task/state related components are decomposed from the multichannel data using mathematical signal processing techniques, such as ICA, PCA, SSD, and CSP. Each component *z*_*i*_(*t*) is obtained from the channel signals **x**(*t*) by applying a spatial filter **w**_*i*_: $z_{i}\left(t\right)={\text{w}}_{i}^{T}\text{x}\left(t\right)$. This way, functionally relevant NFB can be generated without the need to solve the complete inverse problem. Individualized spatial filters constructed by NFBLab reflect activity of specific neuronal populations corresponding to the physiological functions of interest. Thus, for example, using the spatial filter obtained by means of the common spatial patterns (CSP) analysis contrasting the eyes-open and eyes-closed conditions, one can derive a spatial filter for extracting activity of the occipital alpha-rhythm generators. A spatial filter for extraction of the sensory-motor rhythm can be obtained using SSD analysis of the data collected during motor relaxation.

Spatial filtering can be used not only to extract functionally relevant signals but also to suppress the irrelevant signals ubiquitously present in the neural recordings. These are artifacts resulting from the heartbeat, eye blinks and other sources, and/or neural modulations unrelated to the function of interest. The irrelevant signals are found after computing a spatial decomposition of the EEG/MEG data and examining the component time-series $\text{z}\left(t\right)={\left[z_{1}\left(t\right),z_{2}\left(t\right),\ldots ,z_{N}\left(t\right)\right]}^{T}=\text{B}\text{x}\left(t\right)$, where **B** is spatial decomposition matrix (e.g., unmixing matrix in case of ICA). For example, the *k*-th component reflects an artifact and needs to be removed. This could be done by simply zeroing the *k*-th component *z*_*k*_(*t*) = 0 and reconstructing the data from the rest of the components. This is equivalent to excluding the *k*-th row in the analysis matrix **B** and removing the *k*-th column of matrix **C** = **B**^−1^. The denoised data can be represented as ${\text{x}}_{c}\left(t\right)={\text{B}}_{-k}^{-1}{\text{B}}_{-k}\text{x}\left(t\right)$ where the subscript “−*k*” indicates the removal of the *k*-th column and the *k*-th row from **B**^−1^ and **B**, respectively. Such data cleaning can be combined with the spatial filtering operation described above. The spatial filter takes the form ${\bar{\text{w}}}_{i}={\left({\text{B}}^{-1}\right)}_{-k}{\text{B}}_{-k}{\text{w}}_{i}$ and then the interference-free virtual derivation signal can be computed as $y_{i}\left(t\right)={\bar{\text{w}}}_{i}^{T}\text{x}\left(t\right)$.

### 4.2. Narrow-Band Component Envelope

Many NFB applications are based on the estimation of dynamical changes in oscillatory brain activity, such as EEG rhythms in specific frequency bands. Brain rhythms of interest are extracted from the ongoing EEG/MEG activity using digital band-pass filters. Filter parameters are calculated based on the frequency range and filter order specified by the user. Generally, rhythmic brain activity is non-stationary and can be quantified as the instantaneous power of the oscillatory component envelope. In what follows we will denote as *d*_*i*_(*t*) the envelope of the narrow-band virtual derivation signal *y*_*i*_(*t*). Signals *d*_*i*_(*t*) are then referred to as *derived signals*.

#### 4.2.1. Basic Methods

**Figure 3:**
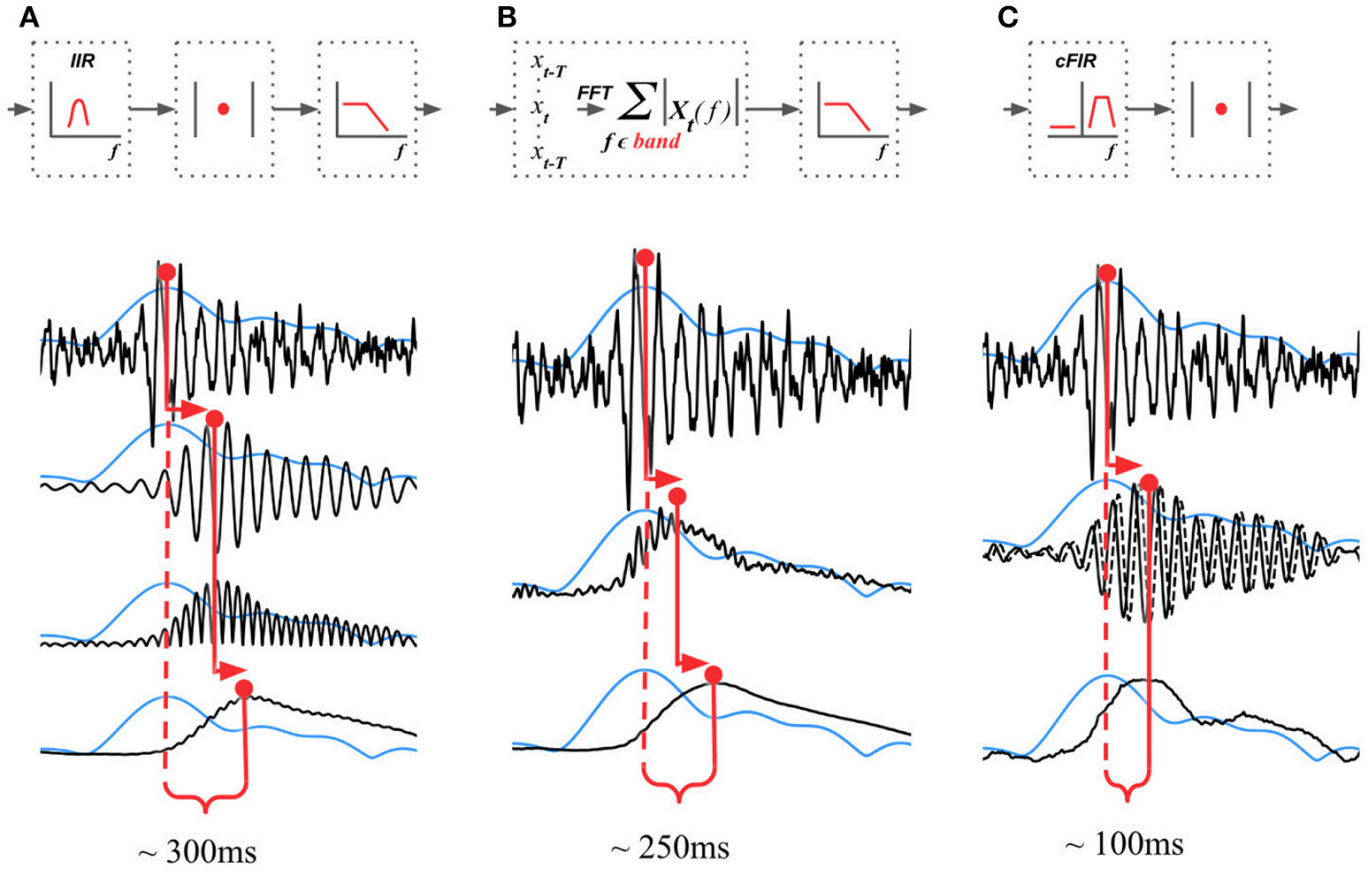
Techniques that can be used to estimate the envelope of a narrow-band signal. **(A)** Narrow-band filtering with an infinite impulse response (IIR) filter followed by the rectification results in a significant delay. **(B)** STFT with subsequent temporal smoothing of the absolute values of the STFT coefficient. Combined with mirror-reflection, this technique allows to slightly reduce the delay as compared to **(A)**. **(C)** Complex demodulation based technique that has the lowest delay and allows to obtain narrow-band envelope estimation with the latency of 100 ms. The typical delay values reported for each of the techniques correspond to the combination of parameters that deliver comparable accuracy of narrow-band envelope estimation with all the three methods.

**Figure 4:**
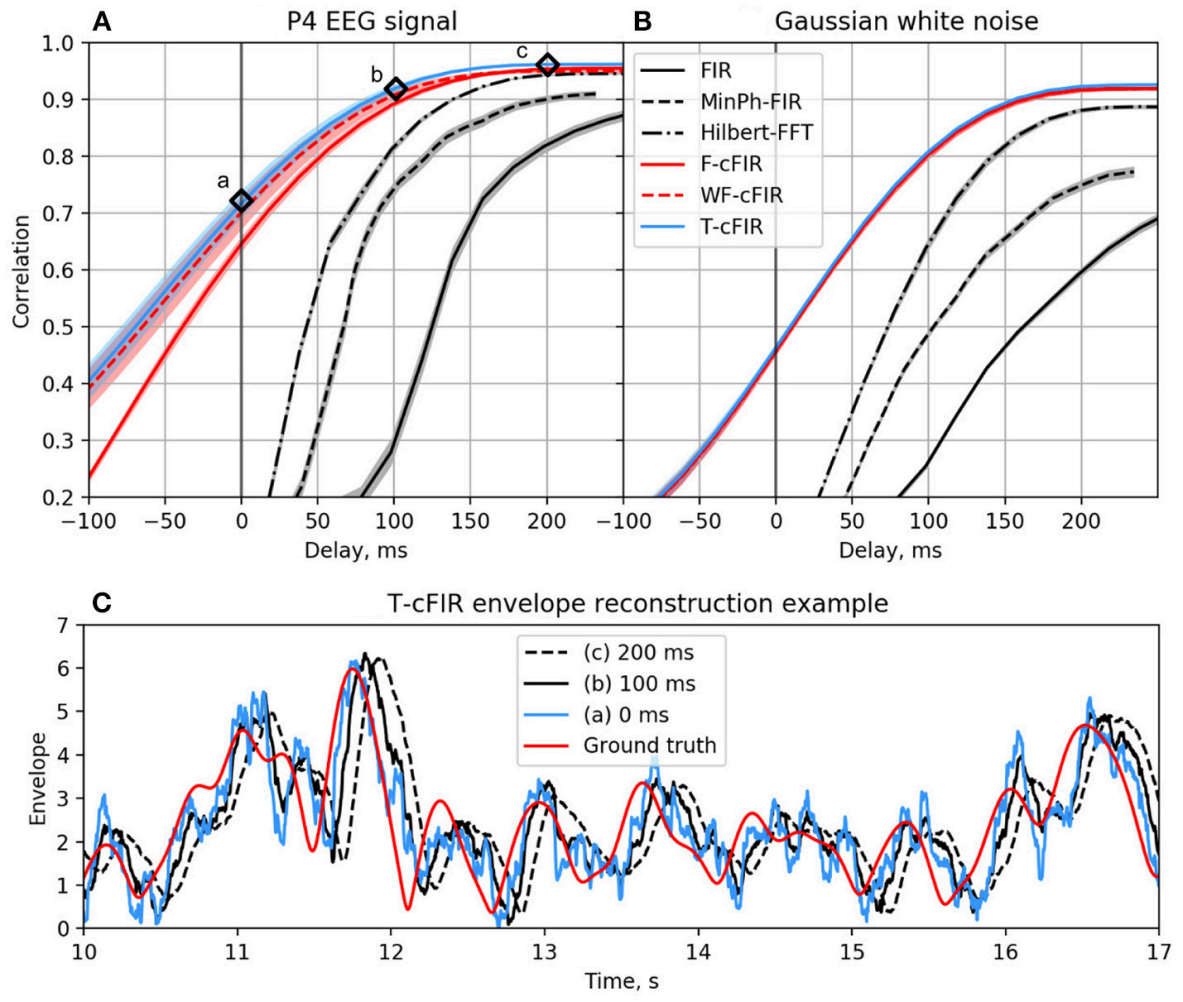
Correlation coefficient for the envelope obtained causally by each of classic (black curves) and novel (colored curves) filters with the ground-truth sequence vs. filters group delay for real EEG P4-channel data **(A)** and white noise signal **(B)**. Example of T-cFIR envelope reconstruction for 0, 100, and 200 ms delays **(C)**.

The existing NFB implementations use two major methods to estimate the envelope. The first method consists of performing low-pass filtering of the rectified narrow-band signal. This method is represented by the diagram in Figure 3A. This simple and ubiquitously used approach, however, introduces considerable delays that make it difficult for the subject to utilize the resultant NFB as it often arrives when the reinforced activity pattern has been already abandoned. Consequently, the desired pattern of brain activity cannot be efficiently reinforced. Attempts to reduce the delay using lower-order filters (including their minimum phase versions) result in a rapid deterioration of performance as shown in Figure 4 by the dashed black curve.

The second commonly practiced way to estimate instantaneous band power modulation is illustrated in Figure 3B. This method is based on the use of the short-time Fourier transform (STFT) with subsequent temporal smoothing of the absolute values of the STFT coefficients. We suggest that the temporal performance of the STFT-based technique can be improved with the approach where the data segment is mirror-reflected against the current time, followed by windowing and Fourier transform. Therefore, the FFT is applied to the symmetric sequence created from the segment of the last *T* samples reflected around the last acquired time-sample, which yields *X*_*t*−*T*_, *X*_*t*−*T*+1_, …, *X*_*t*_, …, *X*_*t*−*T*+1_, *X*_*t*−*T*_ as a sequence to perform the FFT on. This way, the subsequent application of a symmetric scaling window (Hann, Hanning, Blackman, etc.) effectively emphasizes contribution of the most recent samples. This method, on average, shortens the delay to around 250 ms while retaining sufficient accuracy of the envelope profile estimation. As it is the case with the rectification-based method, further attempts to reduce the delay lead to a significant drop in estimation accuracy, as illustrated by the dot-dashed black curve in Figure 4A.

#### 4.2.2. Advanced Envelope Estimation Techniques

In addition to these two methods, NFBLab implements several advanced algorithms for decreasing the delay. An optimal latency-accuracy trade-off can be achieved with these methods. The general idea behind these techniques is based on the use of a finite impulse response filter (FIR) approximation of narrow-band Hilbert transform. This approach is schematically presented in Figure 3C. The accuracy of envelope estimation is inversely proportional to the length of the analysis interval while the longer intervals lead to longer delays. In contrast to the traditional methods, these advanced techniques allow to explicitly specify the desired delay value and then achieve the best possible accuracy for the specified delay.

In the Hilbert transform-based methods, narrow-band signal *s*[*n*] is represented as the real part of the analytic signal:

$$y\left[n\right]=s\left[n\right]+js_{h}\left[n\right]=A\left[n\right]e^{j\left(w_{c}n+\phi \left[n\right]\right)}$$

where *s*_*h*_[*n*] is the imaginary part of the analytic signal, often called “second quadrature” of the original signal *s*[*n*], *w*_*c*_ is the central band frequency, *A*[*n*] is the instantaneous power of the narrowband process, and *ϕ*[*n*]—is the instantaneous phase. Thus, the envelope power *A*[*n*] can be computed as the squared norm of the analytic signal.

To build the analytic signal *y*[*n*] corresponding to the original narrow-band signal *s*[*n*] derived from the noisy broad-band signal *x*[*n*], one can apply a narrow-band Hilbert transform filter. Frequency response of the filter can be defined as:

(1) $$H_{d}(e^{jw})=\{\begin{array}{l} 2e^{-jwd},w\in [w_{c}-\delta w,w_{c}+\delta w]\subseteq [0,\pi ]\\ 0,otherwise \end{array}$$

where δ*w* is the half of the bandwidth and *d* is the group delay in the samples. For any finite *d* this filter is non-causal and cannot be applied in real time. To reconstruct analytical signal causally, one can use a complex-valued finite impulse response filter (cFIR) that approximates frequency response $H_{d}\left(e^{jw}\right)$ . The desired filter **b** can be found by solving the least squares optimization problem defined in different ways.

The first and the most straightforward approach (denoted F-cFIR) that implements the least squares filter design strategy is to find the complex valued vector of cFIR filter weights **b** that minimizes the *L*_2_ distance between the cFIR filter frequency response and the ideal response *H*_*d*_ in the frequency domain:

bF=arg minb||Fb-Hd||L2

where *F* is the discrete Fourier transform operator. This simple approach does not take into account the EEG temporal structure and can be further improved.

One improvement can be achieved using optimal filter design ideas. In this approach, spectral density *X* of the input signal *x*[*n*] provides weights. We thus formulate the weighted frequency domain least squares design technique (denoted WF-cFIR) as the following optimization problem :

bWF=arg minb||(Fb-Hd)·X||L2

This method allows to exploit the patterns and hidden relationships between rhythmic components in the EEG signal.

The last approach from this family (denoted T-cFIR) is based on minimization of the squared distance in the time domain between the complex delayed ground truth signal *y*[*n*−*d*] and the filtered signal *x*[*n*]*b[*n*]:

$${\text{b}}_{T}=arg\underset{\text{b}}{\min}||x\left[n\right]*\text{b}\left[n\right]-y\left[n-d\right]|{|}_{L_{2}}$$

where * is the convolution operator. Here, during the training stage, the ground truth signal *y*_*d*_ is obtained non-causally from the training dataset via ideal zero-phase Hilbert transformer (1). According to Parseval's theorem, this approach is equivalent to the WF-cFIR approach. However, in contrast to the frequency domain formulation, this method uses a straightforward way to add amplitude dependent weights in order to achieve better accuracy of the envelope shape estimation.

As illustrated in Figures 4A,C the advanced techniques described above allow for envelope estimation with a significant improvement in latency and accuracy as compared to standard methods (colored vs. black lines) used in the majority of the NFB software. When applied to a white noise sequence, see Figure 4B, the performance of envelope estimation methods tends to decrease.

It has been recently emphasized that both beta-rhythm (Shin et al., 2017) and alpha-rhythm activity (Ossadtchi et al., 2017) can be described as a sequence of oscillatory episodes, i.e., discrete events rather than continuous modulations of these rhythms. During alpha NFB training, the duration of each oscillatory episode stays constant, and the training effect consists of the increase in the number of episodes per unit time (Ossadtchi et al., 2017). These events (e.g., beta or alpha episodes) have characteristic duration of 300 ms or shorter. Given such a short duration, the decreased NFB latency achieved with the advanced techniques will result in reinforcement arriving on time, during the rhythmic episode and not when it has finished (Oblak et al., 2017).

### 4.3. Normalization and Standardization

It is customary for NFB applications to apply various normalization methods prior to presenting NFB to the subject (Enriquez-Geppert et al., 2017). In NFBLab, derived and composite signals are standardized based on their statistics estimated for a baseline data segment. Standardization consists of subtracting the mean of the derived/composite signal *y*(*t*) and division by the standard deviation: $ȳ\left(t\right)=\frac{y\left(t\right)-m_{y}}{\sigma_{y}}$. This operation ensures that ȳ(*t*) is centered around zero with maximal amplitude around 3. The use of the standardized signal allows to easily control the fraction of outliers and adjust the feedback threshold (Thatcher and Lubar, 2008). Additionally, median based statistics can be used for robust normalization of outliers.

Some applications require operations with only positive signals. In these cases, robust estimates of *y*_*min*_ and *y*_*max*_ statistics can be used, where normalized signal is computed as $ȳ\left(t\right)=\frac{y\left(t\right)-y_{min}}{y_{max}-y_{min}}$. The normalized signal changes in the [0, 1] range.

### 4.4. Motor Imagery BCI Paradigm Support

The derived signals-based structure fits well into the processing framework of modern motor-imagery BCIs. Accordingly, a separate module of NFBLab software offers a solution for generating the derived signals based on the output of an EEG classifier tuned to distinguish motor-imagery commands. This processing pipeline includes: (1) pre-filtering of EEG signal in some relatively broad frequency range, e.g., 0.5-45 Hz, (2) followed by the band-pass filtering of data in several overlapping bands (e.g., 6–10 Hz, 8–12 Hz, …, 20–24 Hz), (3) spatial decomposition, e.g., CSP aimed at contrasting the data recorded within different motor-imagery states; (4) estimation of the instantaneous power in each of the spatial components isolated at step 3 and smoothing with a moving average (MA) with a time constant of 0.5 s; (5) application of a motor-imagery classifier (e.g., a classifier for delineating right and left hand imagery and resting conditions). Stages 3 and 5 include parameter adjustments for individual users; this adjustment requires an EEG sample recorded during the imagery states of interest.

## 5. Main NFBLab Objects and Their Descriptors

### 5.1. Derived Signal

**Figure 5:**
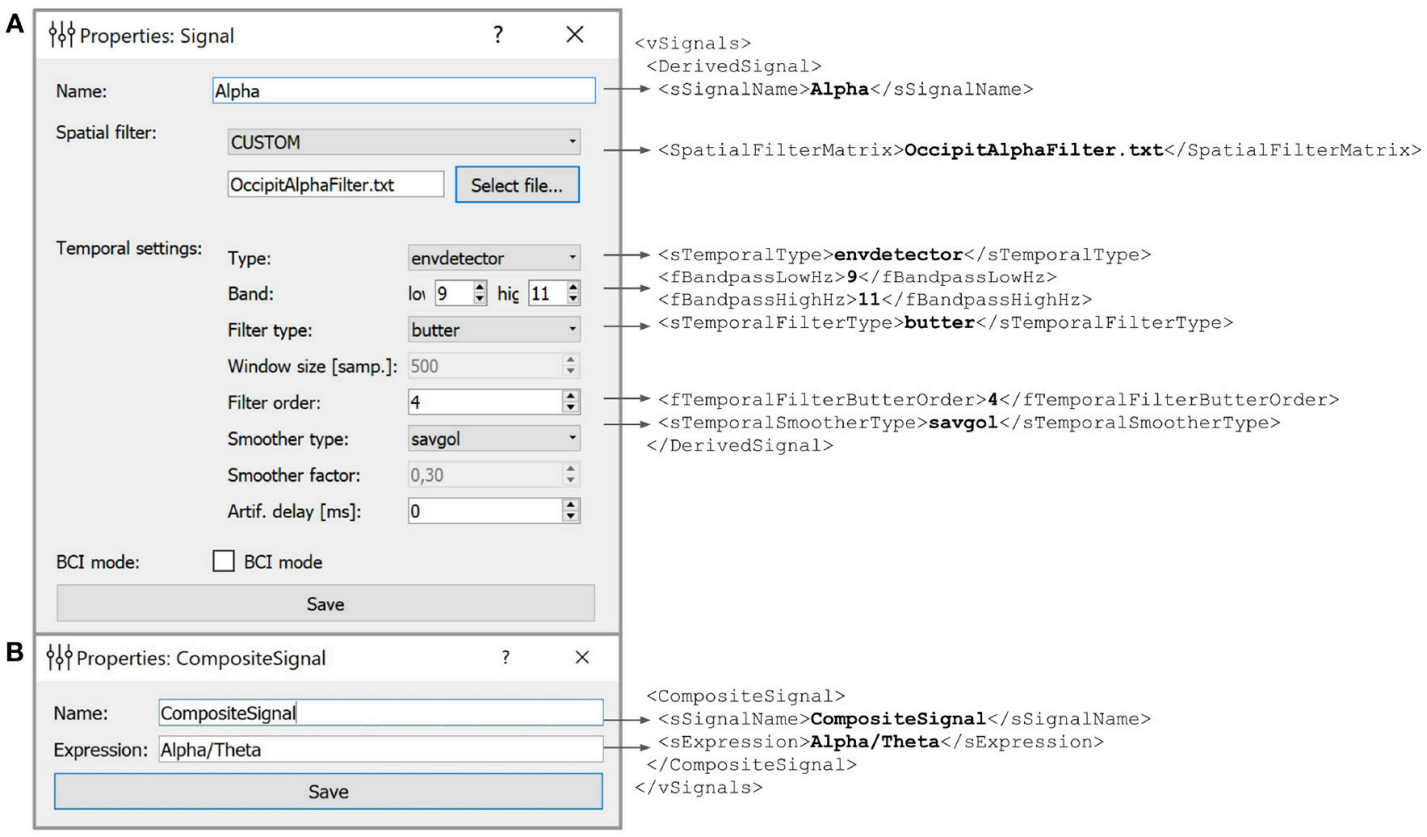
Example of the derived **(A)** and composite **(B)** signals settings along with the XML script lines the GUI based settings are translated to. Derived Signals description is stored in the *vSignals* section of the XML file.

According to the NFBLab terminology, derived signal (DS) is the envelope of the narrow-band filtered virtual lead signal. In most cases, the DSs are scalars varying over time. The number of DSs that a user can define is limited only by the performance characteristics of the computer running the NFBLab. The DSs are described in a specific XML structure. Figure 5A shows the dialog for editing DS settings. This dialog can be called from the “Experiment design” module before launching an experiment. Additionally, DSs can be set and/or changed based on the data from the functional tasks. For this purpose, an interactive *Data-driven filter designer* module can be called during an experiment. This tool allows to choose the desired frequency band, perform spatial decomposition of the data and choose appropriate spatial filters. In addition to the interactive dialogs, advanced users can edit the XML file directly. The DSs can be linked to the NFB presentation module so that NFB output represents one of the DSs or a composite signal formed from several DSs.

In the example shown in Figure 5 we have defined a Derived Signal named *Alpha* to be obtained from the raw EEG data by spatial filter coefficients stored in *OccipitAlphaFilter.txt* and with 4-th order Butterworth signal with a passband of 9–11 Hz. Envelope smoothing is performed with Savitsky-Golay polynomial filtering.

### 5.2. Composite Signal

NFB often incorporates several signals, such as different EEG rhythms. For example, alpha-to-theta ratio feedback is based on the instantaneous power of the alpha band divided by the power of the beta band. For such computations, NFBLab implements the composite signal class, which is defined as an arbitrary mathematical function of two DSs. As shown in Figure 5B, this function is specified by the user. For example, to define a training protocol which requires calculation of the ratio of the occipital alpha rhythm power to the frontal theta rhythm power, the DSs representing the corresponding bands are used, and the division function is specified. NFBLab also allows for more complex computations, such as assessment of correlation between virtual lead signals using such metrics as coherence or envelope correlation.

In addition to forming NFB, composite signals can be used to track the presence of artifacts in neural recordings, such as eye blinks, muscular artifacts, and mechanical artifacts caused by movements. A derived or composite signal representing artifact can be converted into a warning stimulus instructing the subject to correct the undesired behavior.

### 5.3. Experimental Block

NFB experiments usually consist of several blocks. In NFBLab, a block is defined as the task part where the stimulus settings and signal processing procedures remain unchanged. After a block is finished running, the recorded data is stored in the HDF5 file. Next, one or several actions from the following list are executed:

- the mean and the standard deviation are updated, which are then used to standardize the feedback signal, see section 4.3;
- the interactive Data-driven filter design module is launched to modify or create the DSs based on the data from functional tests;
- a sound signal is issued after the block completion.
- the experiment is suspended;

The following block types are currently supported:

- *Baseline*. Baseline blocks are useful for recording background activity or running functional tests. In this block type, either a cross is displayed in the user window or a text message with instructions is shown. The data collected during these blocks are utilized for calculation of the standardization parameters and as an input to the Data-driven filter design module for deriving spatial and temporal filters.
- *Feedback*. This is the main module during which the software displays NFB in the user-selected form. In addition to presenting real NFB, this block supports mock feedback generation. To activate this mode, the user has to specify the name of a prerecorded data file whose data will be used for calculation of the feedback signal. Additionally, to implement mock feedback condition, one can configure the software to use the data file recorded during one of the previous blocks.
- *Video*. This block type allows a video (specified in the block settings) to be played on the screen.
- *Trials*. This block type is used in the experiments recording stimulus evoked potentials. On each trial, a stimulus appears on the screen, and the brain response to the stimulus is measured. Different stimuli can be arranged in a predetermined order or randomized. Stimulus and EEG data synchronization is an important issue. One option for syncing the stimuli and the other signals being recorded is to have the stimulus accompanied by a change of brightness within a limited area in the screen corner that is then detected by a photo-sensor attached to the screen. The sensor signal is then fed into one of the auxiliary channels (see also section 7). Stimuli identifiers and timestamps are saved to the data file.
- *Center*-*Out*. This block type implements the center-out task (Georgopoulos et al., 1982). The block consists of a sequence of events, such as appearances of screen targets at the central and peripheral positions. The subject moves a cursor (using a joystick or mouse, or through BCI control) from one target to another. The stimuli are synced with the other signals in the data stream. The identifiers and timestamps of all the events are saved to the data file.

Based on these examples, new block types can be added to NFBLab, including the ones that require external visualization programs.

**Figure 6:**
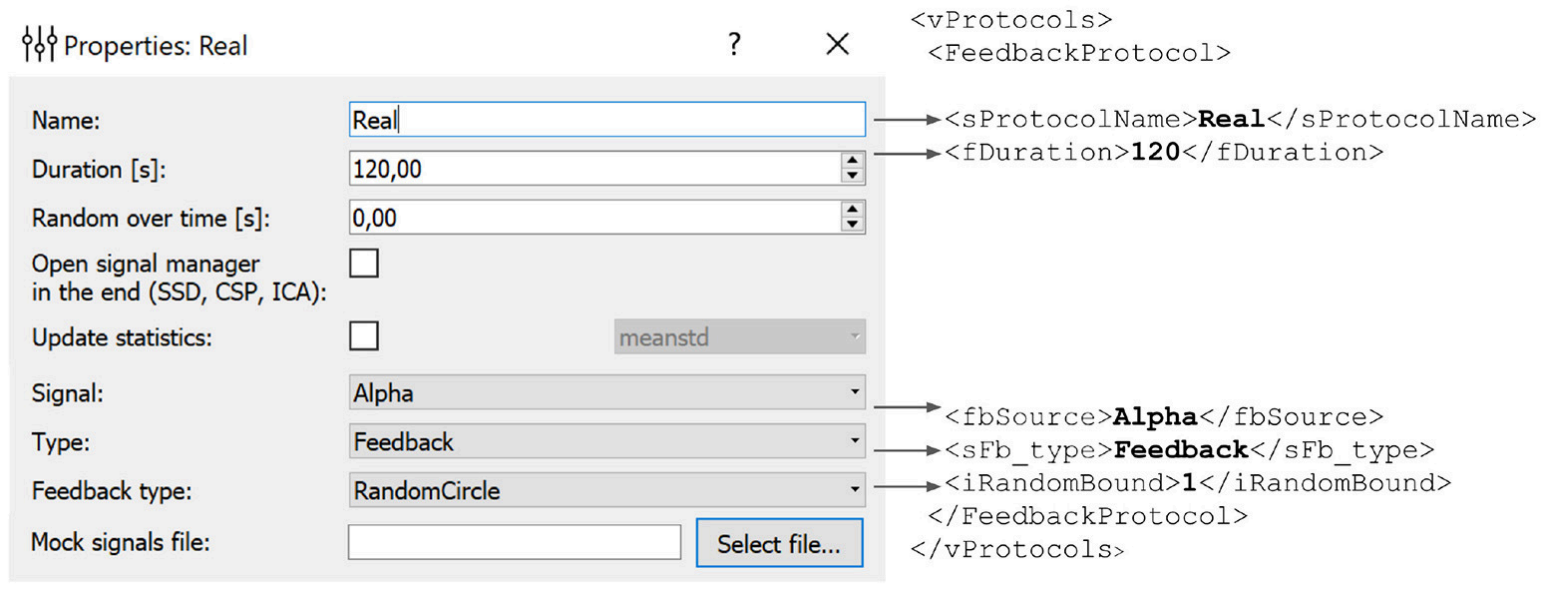
Experimental block settings can be adjusted using the GUI. The parameters specified in the GUI are translated into the corresponding portion of the XML file. Description of each block is stored in the *vProtocols* section of the XML file. Here we defined a block named *Real* of type *Feedback* with feedback display in the form of a circle with uneven boundary shape. After the protocol is finished the statistics (mean and standard deviation) will not recalculated. Block duration is set to 120 s.

In the example presented in Figure 6, we define an experimental block named *Real* of type *Feedback* that will display feedback in the form of a circle with non-harmonic (random) boundary shape. The protocol will not result in an update of the statistics (mean and standard deviation) used for normalization of the feedback signal. The block will last for 120 s.

### 5.4. Experimental Protocol

**Figure 7:**
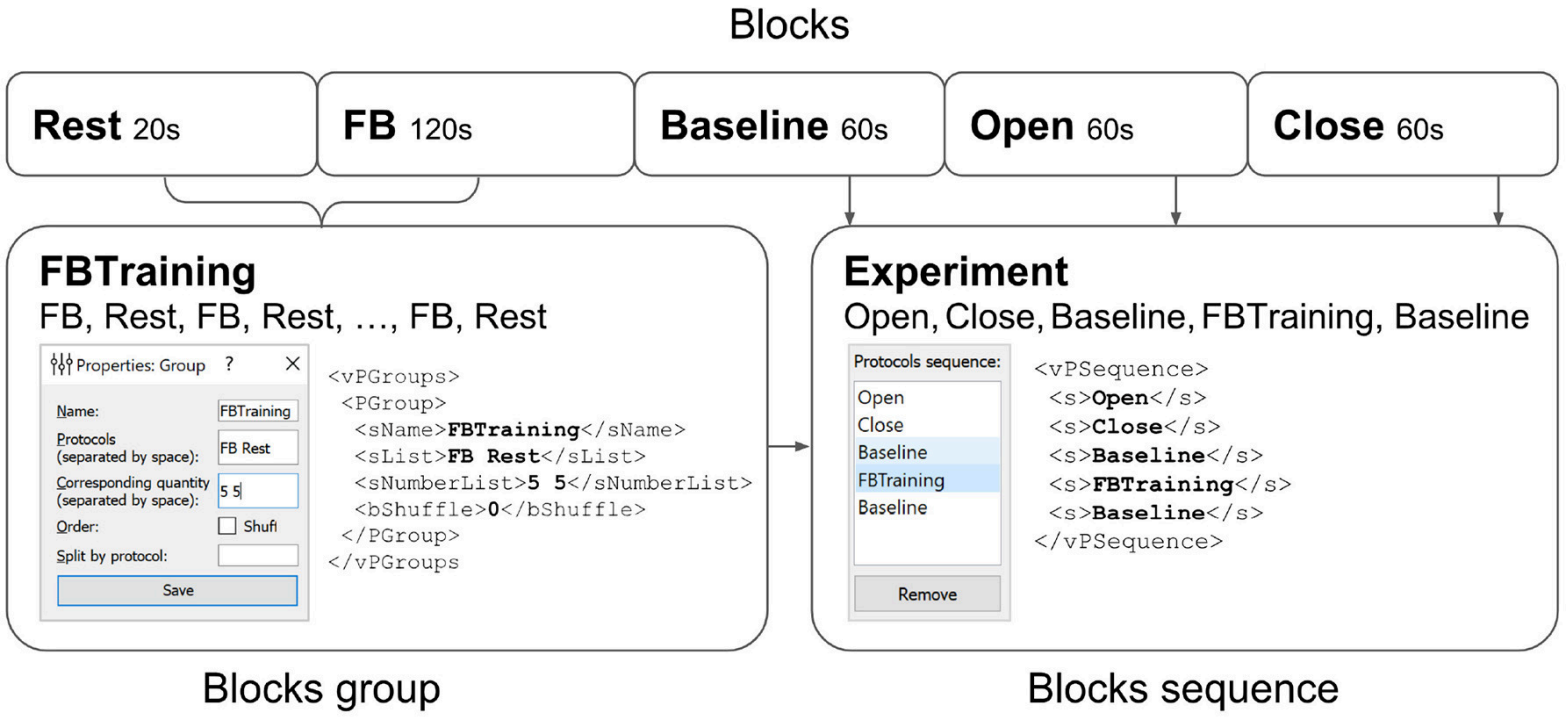
Experimental protocol example. A group of experimental blocks called *FBTraining* is defined to consist from of the actual feedback (*FB*) and rest (*Rest*) blocks. The repetition count for this unshuffled pair is set to 5. The actual sequence of blocks to be performed is then specified in the *vPSequence* section of the XML file. As shown in the right panel the experiment itself comprises blocks (*Baseline*,*Open*,*Close*). XML file reflects this construction within *vPSequence* section.

The overall experimental protocol is defined by the sequence of blocks entered in the configuration settings (Figure 7). Upon the completion of each block, one or several events are executed, as explained in the previous section.

It is also possible to define groups of experimental blocks that consist of several blocks to be executed in a specific order. It is also possible to define randomization within each of the groups. A group of experimental blocks can be set up from the corresponding GUI and will be reflected in the *vPGroups* section of the XML file. In Figure 7, we define an experimental blocks group called *FBTraining* that consists of the actual feedback (*FB*) and rest (*Rest*) blocks. This sequence of two blocks will be repeated 5 times in this particular order (first *FB*, then *Rest*). The actual sequence of blocks to be performed is then specified in the *vPSequence* section of the XML file. In this example, the experiment comprises blocks (*Baseline*,*Open*,*Close*) and the above defined block group, as illustrated in the right panel of the figure. XML file reflects this construction within *vPSequence* section.

It is also possible to define a group of blocks with random shuffle requirement. In this setting, block order will be randomized and the rate of appearance for block will be proportional to the specified value.

All experimental protocol settings can be specified using NFBLab's Experiment protocol editor, implemented as GUI (described next).

## 6. NFBLab Modules

### 6.1. Experiment Protocol Editor

**Figure 8:**
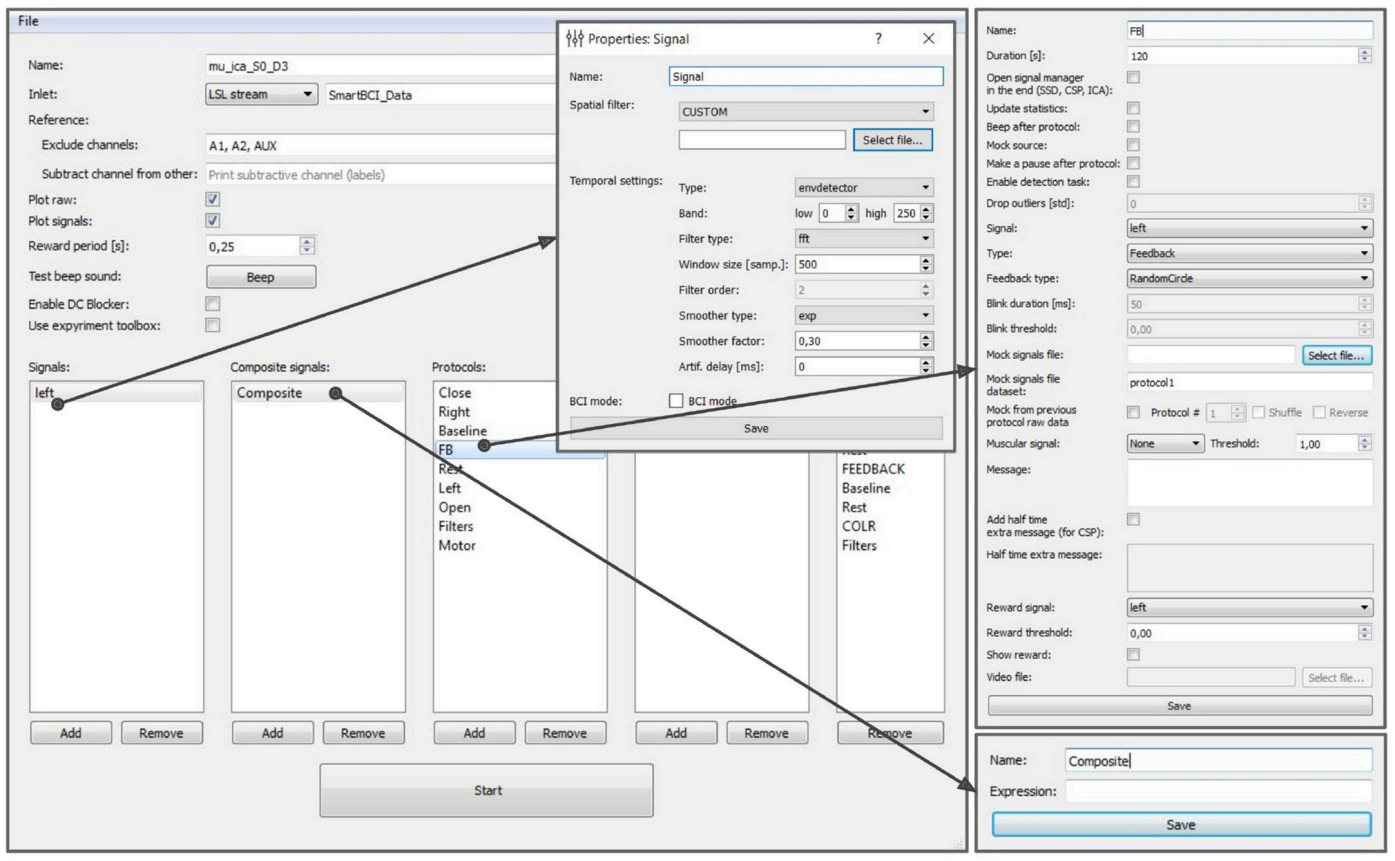
Graphical user interface of the experiment protocol editor. This interface provides access to all the GUI components described in section 6 and allows to fully configure an experiment.

The experimental protocol can be configured using the graphical user interface (GUI) (see Figure 8). This interface provides access to all the GUI components described in section 6 and allows to fully configure an experiment. The GUI allows to define and modify derived and composite signals, set experimental blocks parameters, organize blocks into groups, and specify the experimental sequence. These settings are stored in an XML file and can be loaded later if a similar experiment needs to be conducted. After the experimental protocol is defined through the GUI or loaded from a file, the Experimental module GUI (described next) can be launched by pressing the Start button in the bottom of the Experiment protocol editor panel.

### 6.2. Experimental Module

**Figure 9:**
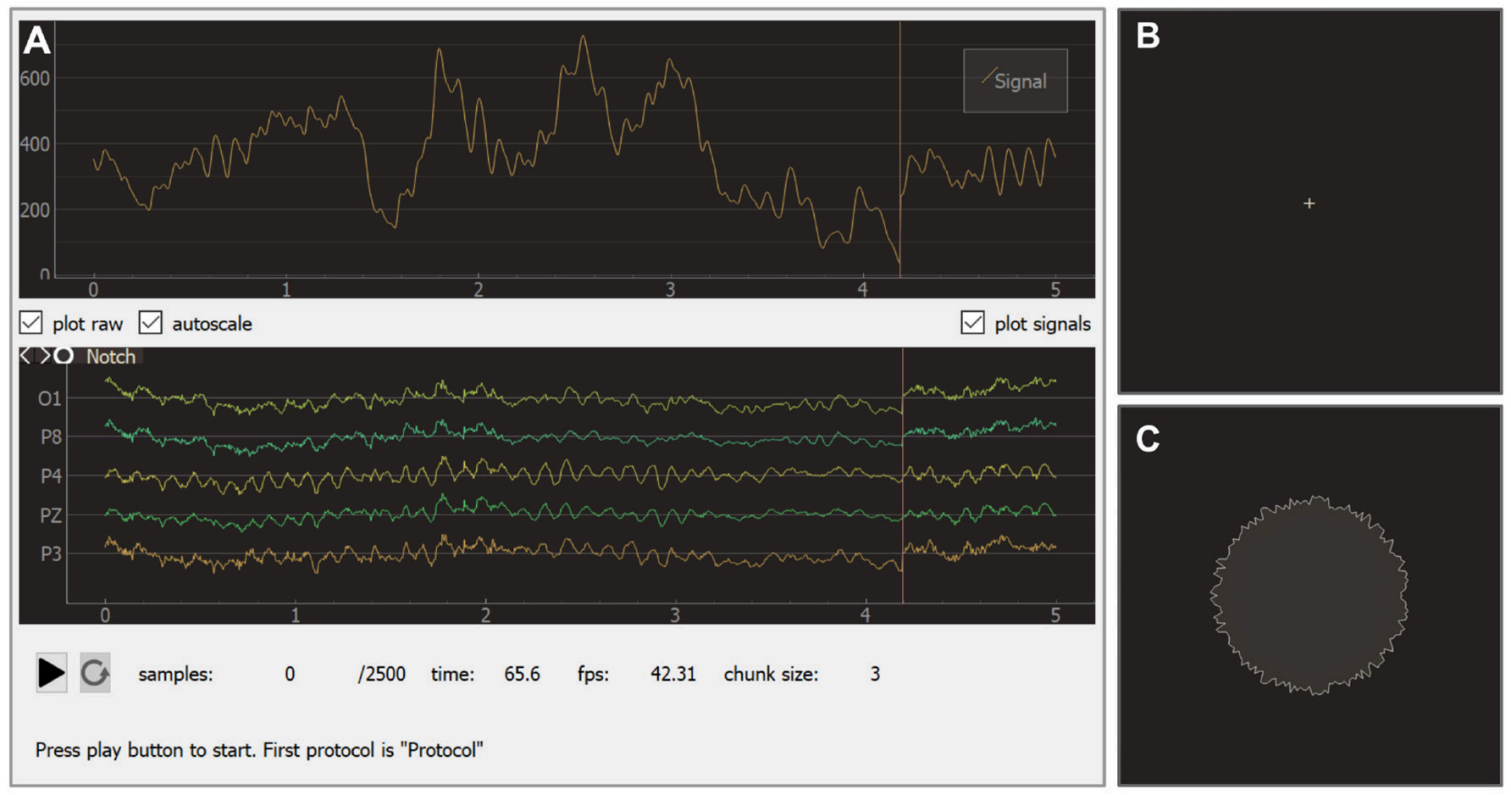
Graphical user interface of the experiment module: experimenter window **(A)**, subject window for baseline **(B)**, and feedback **(C)** blocks.

The experimental module receives raw data from the EEG/ECoG/MEG recording devices, updates all signals, visualizes NFB according to the specifications in each block, controls the sequence of blocks, triggers inter-block events, and saves the experimental data. The experimental module interface (see Figure 9) consists of two main windows: the experimenter's window and the subject's window. In the experimenter's window (Figure 9A) processed signals are displayed in the upper part of the screen and raw signals in the lower part. Textual information about the experiment including the frame rate is displayed at the bottom of the screen. Additionally, the experimenter's window contains start/pause and restart buttons.

During an experiment, the subject faces the monitor that displays the subject's window. Depending on the current block, this window may show different visual stimuli or instructions. The sequence usually starts from a baseline block with a cross displayed in the screen (see Figure 9B). During the actual feedback session (i.e., block of type *feedback*), a visual indicator of NFB is presented. In the example shown in Figure 9C, the shape of a circle carries NFB information: subjects are instructed to make the circle's boundary smoother by modulating their brain activity.

Raw and processed signals, experimental parameters, signal and block properties are saved as an HDF5 (Hierarchical Data Format) file. This data storage format is widely supported by various libraries, including the ones implemented in Python (h5py package), Matlab (built-in function hdf5read) and R (package h5). The detailed structure of the experiment file can be found in an on-line file^5^.

### 6.3. Data-Driven Filters Designer

**Figure 10:**
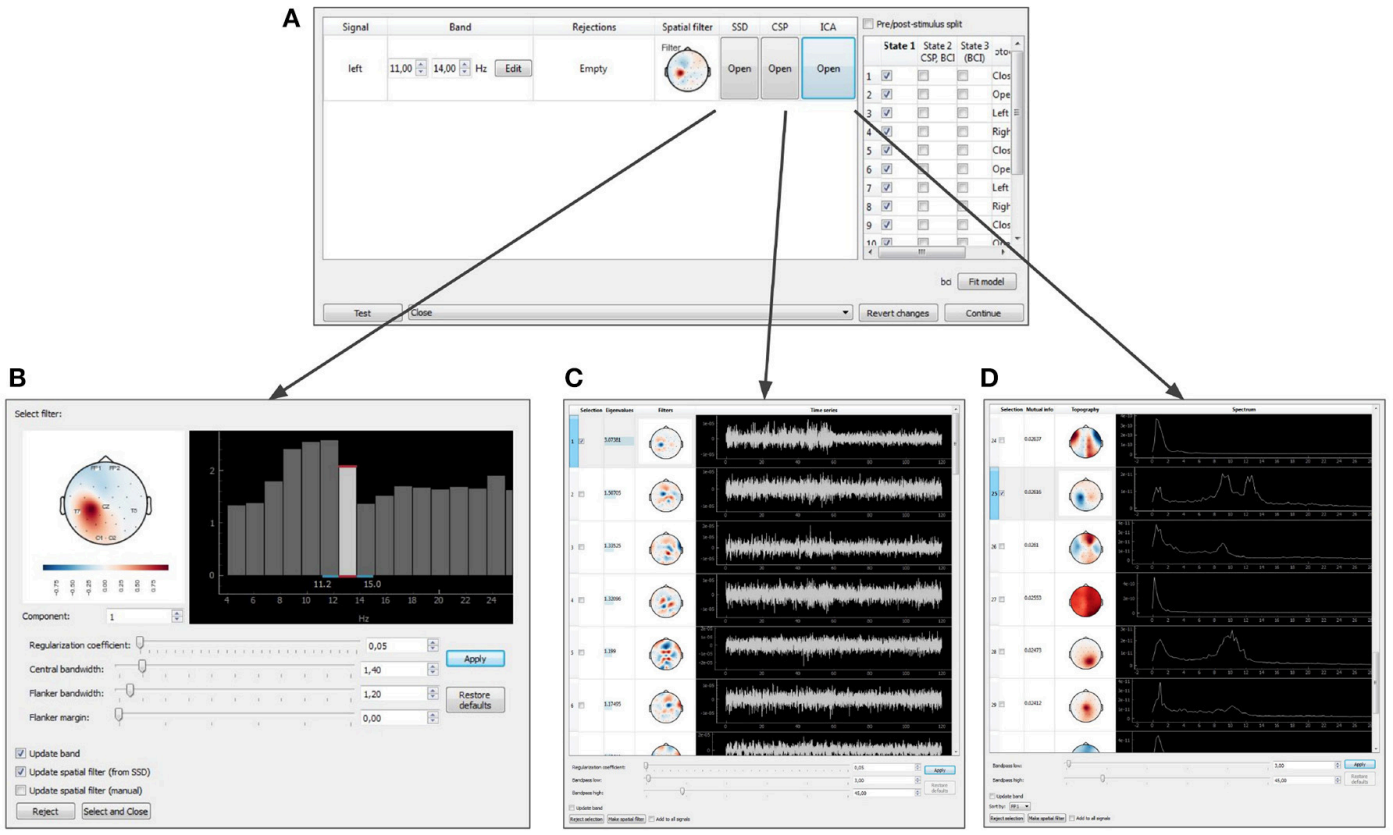
Graphical user interface of the data-driven filter designer: signals manager **(A)**, SSD toolbox **(B)**, CSP toolbox **(C)**, ICA toolbox **(D)**.

After the baseline data is collected, NFBLab offers to adjust the signal processing pipeline based on the functional tests. The temporal and spatial filters can be adjusted interactively using the Data-driven filters designer module. The adjustment can start, for example, with an interactive spectral analysis (see Figures 10A,D) that allows to fine-tune EEG frequency bands and redesign the temporal filter to make it, for example, a better match to subject's individual alpha range. Next, spatial decomposition techniques (ICA,SSD or CSP) (see Figures 10A,B) can be invoked to decompose the data into spatial components that represent both functionally relevant neural modulations and artifacts. Using a selector sub-menu (see Figure 10C), it is possible to specify spatial filters that correspond to the features of interest (e.g., sensorimotor rhythm, alpha-rhythm, etc.), and pin-point the spatial components representing the artifacts to be removed. Different methods of spatial decomposition are available within this module.

Independent Component Analysis (ICA) decomposes the multichannel signal into the components representing the functionally relevant modulations and the artifacts (Bell and Sejnowski, 1995). Common Spatial Pattern (CSP) selects the components that contrast signal modulations between the pair of conditions of a functional test. The algorithm is based on solving the generalized eigenvalue problem (Koles et al., 1990) formed by a pair of autocorrelation matrices for the two different conditions being contrasted. Spatio-Spectral Decomposition (SSD) method also solves the generalized eigenvalue problem. This method maximizes the ratio of power in two frequency bands (the central band and the adjacent flanker sub-bands), and allows to find spatial filters that emphasize narrow-band oscillatory activity (Nikulin et al., 2011).

When the filter configuration module is called, the experiment pauses and the target signal manager opens (Figure 10A). At this point, parameters of spatial and temporal filters are shown and can be edited for each DS. As explained above, a spatial filter combines a notch matrix and a filter vector, and converts a multidimensional signal into a scalar. The filter parameters can be edited manually or determined with one of the methods described above (ICA, CSP, or SSD). For each type of analysis, the user can select the components to remove (the notch matrix maps input data into the space orthogonal to the components containing artifacts) and the components to contribute to NFB. The components are selected based on their scalp topography and patterns in the temporal and frequency domains.

## 7. Feedback Presentation Delay Control

**Figure 11:**
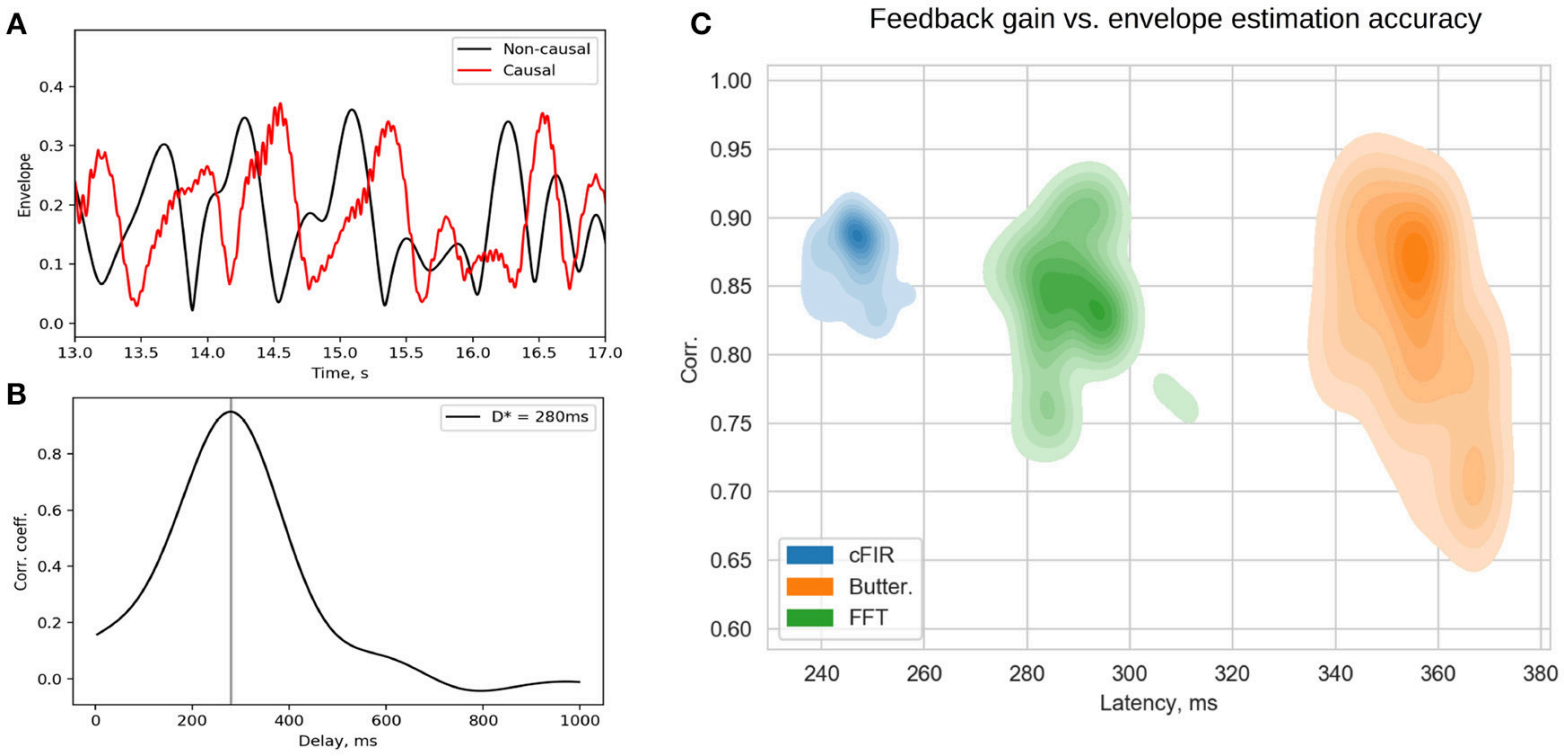
Estimated *p*(*t*) and ideal non-causally estimated envelope *y*_0_(*t*) **(A)** are separated by a lag that can be assessed using the cross-correlation between *p*(*t*) and *y*_0_(*t*) **(B)**. Joint distribution of envelope estimation accuracy (maximum value of the cross-correlation sequence) and total feedback presentation delay (lag of the cross-correlation sequence) for the three different methods **(C)** as estimated from a large number overlapping windows.

Rapid presentation of NFB is the distinctive feature of the NFBLab software. To allow for continuous control of visual feedback presentation latency the NFBLab software supports the photo-sensor based loop. We use small square in the upper-right screen corner whose intensity is modulated by the actual feedback signal presented on the main portion of the same screen. The observed within this square intensity fluctuations are registered by the photo-sensor and fed to one of the auxiliary channels of the EEG device to be registered as *p*(*t*). When an appropriate photo-sensor has used the shape of *p*(*t*) closely matches the corresponding derived signal *y*(*t*) used as a feedback signal. Obtained in real-time conditions under causal restrictions signal *y*(*t*) is a delayed version of the ideal derived signal *y*_0_(*t*) that can be calculated offline by using zero-phase batch filtering. Thus, signal *p*(*t*) appears to be a slightly distorted version of *y*(*t*) and is delayed by some specific value with respect to *y*_0_(*t*). This lag can be determined by computing the estimate of cross-correlation function $R\left(\tau \right)=\frac{E\left\{p\left(t\right)y_{0}\left(t-\tau \right)\right\}}{\sqrt{E\left\{p^{2}\left(t\right)\right\}E\left\{y_{0}^{2}\left(t\right)\right\}}}$ and finding the lag τ_*max*_ corresponding to the maximum value *R*_*max*_ of *R*(τ). Figure 11A shows the two signals *p*(*t*) and *y*_0_(*t*) and Figure 11B illustrates the cross-correlation function. The lag estimated this way represents the true average total delay between the neuronal event and the moment it is reflected in the feedback presented to the subject. Based on the above considerations it is possible to compute a single pair (*R*_*max*_, τ_*max*_) from the data recorded from the entire duration of the experiment in order to assess the average accuracy and latency of the feedback signal. However, given non-stationary nature of the EEG, a more informative approach is to use a sliding window based approach, so that for each data window one can obtain a pair of values characterizing the accuracy and the delay of the feedback that pertains to it and plot the joint distribution of this pair of values over accuracy × latency plane for many possibly overlapping windows. Example of a joint distribution of (accuracy,latency) pairs for the three different envelope estimation methods implemented within NFBLab is shown in Figure 11C. We have chosen the parameters of the three methods to equalize mean envelope estimation accuracy (y-axis). As we can see the Butterworth filter based approach yields the worst accuracy-delay combination as well as the largest variation in the envelope estimation accuracy as compared to the cFIR and the modified windowed FFT techniques. It is also possible to do such computation in a lagged online manner in order to continuously monitor the delay and accuracy parameters of the feedback presented.

## 8. Examples of NFBLab Protocols

All participants of experiments bellow provided a written consent approved by The Higher School of Economics Committee on Interuniversity Surveys and Ethical Assessment of Empirical Research in accordance with the Declaration of Helsinki.

### 8.1. NFB Paradigm Based on the Occipital Alpha Rhythm

Perhaps the simplest NFB implementation is the one that utilizes the prominent occipital alpha rhythm. Commonly, this kind of neurofeedback is performed on the basis of activity in a single EEG channel. Rhythm power is calculated using Fourier transform of 500–1,000 ms data-chunk preceding the feedback presentation time instance. This generally adopted procedure lacks spatial and temporal specificity and could be sub-optimal in terms of training efficiency induced. NFBLab implementation of this simple paradigm addresses both spatial and temporal specificity issues raised above.

To improve spatial specificity we use functional localization approach under which we record and analyze a segment of data collected within the eyes-closed condition and contrast it against the EEG data from the eyes-open condition. Aided with NFBLab interactive tool, we perform CSP spatial decomposition (ICA and SSD are also available) and choose the component with topography corresponding to the occipital generator of the alpha rhythm. This also allows focussing on the alpha frequency band specific to the individual subject. Additionally, the ICA can be performed with an interactive tool (as described in sections 4.1 and 6.3) that chooses spatial components corresponding to the artifact sources to be rejected.

In this example, the band-pass filter is set to the 9–11 Hz frequency range. The envelope detector comprises complex demodulation followed by smoothing with the 2-nd order Savitzky–Golay filter using the 151 samples long window. The resulting delay of envelope estimation (end-to-end) appears to be 131 ms achieved at sampling frequency 250 Hz. The reconstructed envelope matches the ideal Hilbert transform based envelope with an accuracy of 0.7, measured as correlation coefficient. In contrast, FFT and Butterworth filter based approaches, see Figures 3A,B operating at the same accuracy would result into 250–350 ms delay. To further reduce feedback presentation latency and improve temporal specificity of neurofeedback, the novel adaptive complex demodulation approach could also be used (see Figure 3C).

**Figure 12:**
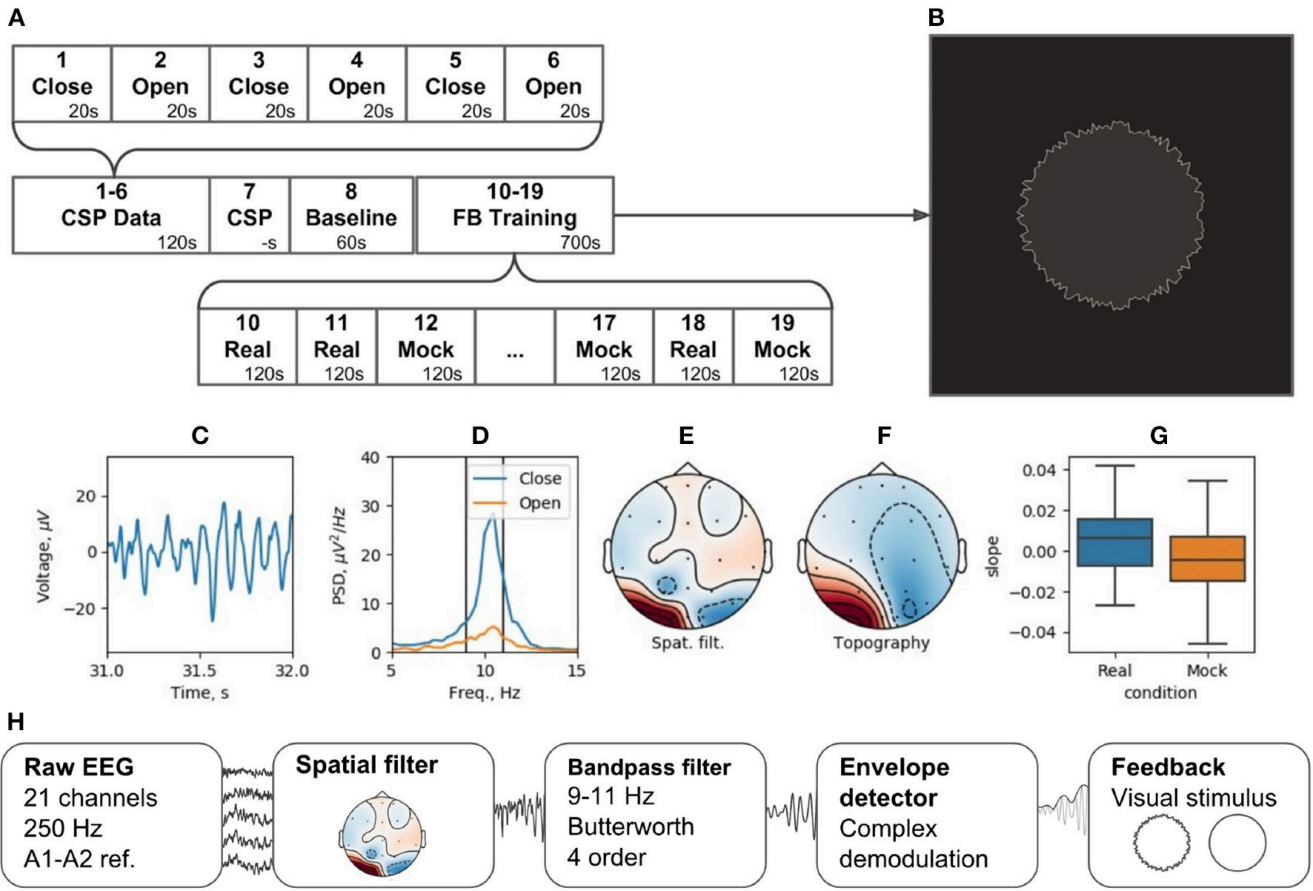
Neurofeedback training experiment example: **(A)** experimental protocol, **(B)** feedback stimulus, **(C)** CSP-component time series segment, **(D)** CSP-component power spectral density (PSD) for closed and open eyes probes, **(E)** CSP-component spatial filter, **(F)** CSP-component topography, **(G)** learning rate in two conditions (Real and Mock feedback), **(H)** signal processing diagram.

The experiment is divided into 19 blocks (see Figure 12A). During blocks 1 through 6, subjects open and close their eyes (“Open” and “Close” blocks). Within Block 7 (“CSP”) of the pipeline, the Data-driven filter designer module is called to derive spatial filter to accommodate individual patterns of the occipital alpha rhythm generator and get rid of artifacts. The CSP analysis contrasts the EEG activity during the “Open” and “Close” blocks. This analysis assumes that the alpha rhythm originates from the same cortical source in both cases, and that the rhythm power increases (i.e., alpha synchronization) in the eyes-closed condition and decreases (i.e., alpha desynchronization) when the eyes are opened. Figures 12C–F shows the characteristics of the selected CSP component, including the segment of its time series containing an alpha episode (C), the spectra of this component for the “Open” and “Close” states (D), the spatial filter (E), and their topography (F).

Following the adjustment of the spatial filter, a resting state (i.e., Baseline block) is recorded to obtain EEG parameters (mean and standard deviation) needed for conversion of the signals into standardized quantities by subtracting the mean and dividing by the standard deviation. The standardization is needed to correctly display the NFB (Figure 12B). For these standardized quantities, the sample mean is 0 and the standard deviation is 1.

The NFB is visualized as a circle with rough (uneven) boundary. The stronger is the NFB signal, the smoother is the boundary. NFB training paradigm consists of five experimental sessions separated by 2-min breaks. Next, five sessions are run with mock NFB. The details of the experimental design are available on-line^6^. Figure 12H illustrates the processing diagram. The diagram consists of the standard blocks for this kind of experiments and, in addition, specifies unique features for each block, such as settings of spatial and temporal requirements.

Here, as an example, we describe results obtained with this paradigm from a single subject. EEG recordings were carried out using a wireless amplifier SmartBCI (Mitsar Co., Ltd). We collected EEG with 21 channels according to the standard 10–20 scheme at 250 Hz sampling rate. The digital average ear (A1 + A2)/2 was used as the reference. As shown in the diagram in Figure 12, the overall duration of the experiment was less than 15 min.

To assess NFB efficiency, we analyzed changes in the NFB power over time within the data segments corresponding to the real and mock NFB training conditions. NFB changes were quantified as the slope of the data-points cloud representing average NFB power within a 1-min time window; 500 windows were randomly allocated to conduct a linear regression analysis. The obtained regression coefficients were significantly different for the mock and real NFB (*p* < 0.001, Wilcoxon's test). The average slope was significantly positive for real NFB (*p* < 0.001, Student's t-test, H0-coefficient = 0) and significantly negative for mock NFB (*p* < 0.001, t-test of the Student, H0 - the coefficient is 0). Thus, only during the real NFB sessions, alpha power experienced statistically significant increase.

### 8.2. Motor-Imagery BCI

**Figure 13:**
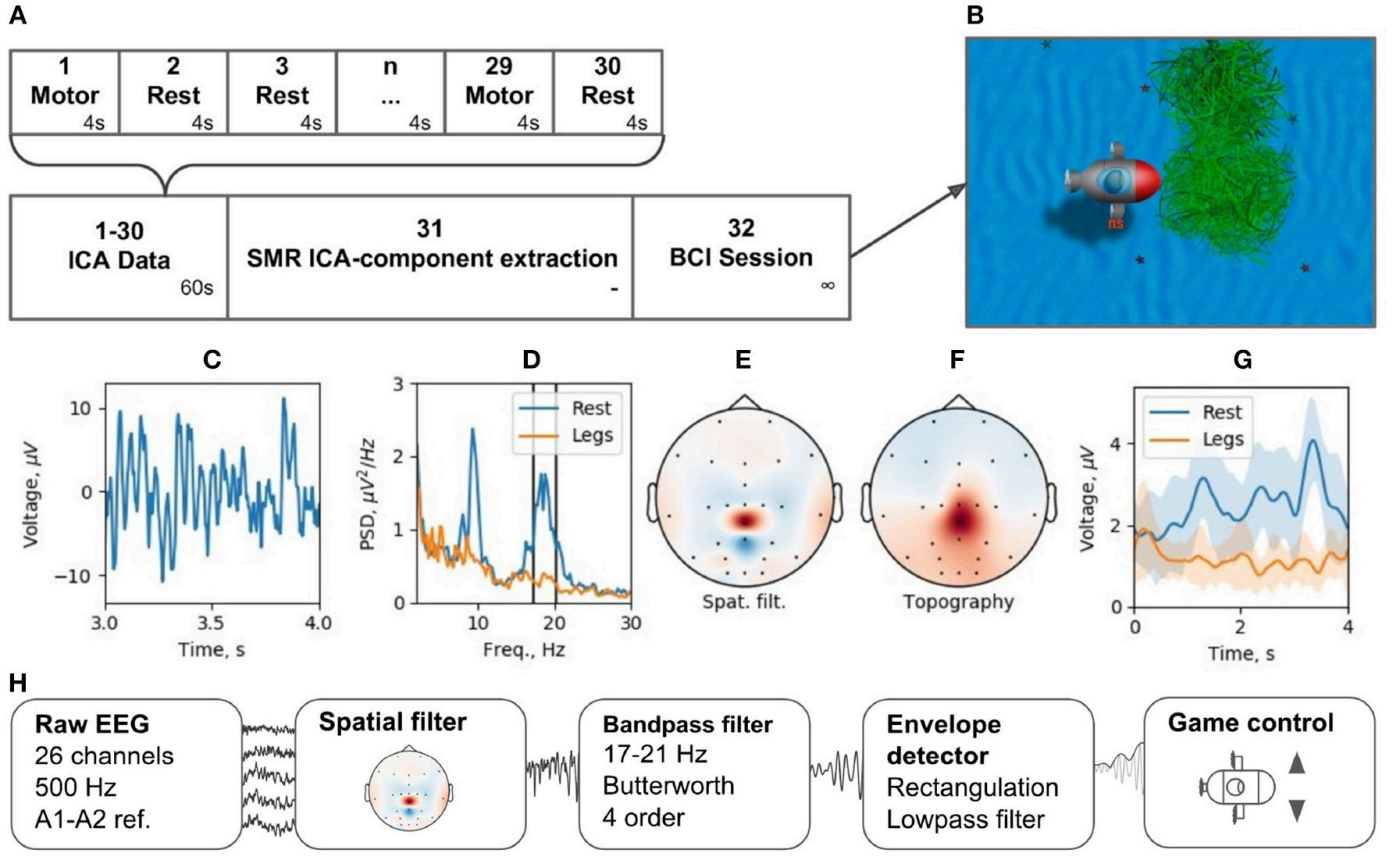
BCI experiment example: **(A)** experimental protocol, **(B)** BCI game, **(C)** ICA-component time series segment, **(D)** ICA-component power spectral density (PSD) for resting state (Rest) and motor task (Legs), **(E)** ICA-component spatial filter, **(F)** ICA-component topography, **(G)** cross-trials averaged envelope in two states (Rest and Legs).

Brain-computer interfaces are another popular real-time EEG paradigm. The described software contains modules that allow for conducting experiments within P300 and motor-imagery BCI paradigms. Making motor-imagery work reliably in patients with neurological and motor deficits is always a challenge that requires individually designed signal processing tracts. In this example, we consider NFBLab implementation of a lower limb motor-imagery BCI (Figure 13) based on the beta components of the patient's sensorimotor rhythm (SMR) power. This signal is derived from multichannel EEG recordings using a combination of individually designed filters. The first processing stage (“ICA Data”) consists of 15 motor-imagery blocks and 15 resting blocks; the duration of each block is 4 s. During a motor-imagery block, the subject imagined limb movements (left hand, right hand, or the legs). Following the collection of this data, “SMR ICA-component extraction” was conducted. The experimenter then used the “Data-driven filter designer” module to perform ICA decomposition of the recorded data and define a spatial filter for extracting SMR. At this stage, it was also possible to adjust the bandwidth that defined SMR and choose the components representing the artifacts to be spatially filtered out from the data. Finally, during the last part of the experiment (“BCI Session”), a BCI loop was implemented for controlling a video game: the output signal was streamed through the LSL interface to a BCI game controlled by two commands: command 1 corresponding to the resting state and command 2 corresponding to the lower limb motor-imagery state.

We tested this experimental protocol at the Neurotlon competition that took place in St. Petersburg, Russia in 2017 (supported by the interdisciplinary union “Neuronet” and the Ministry of Industry and Trade). BCI game consisted of controlling a virtual submarine that had to avoid obstacles by adjusting its position in the vertical dimension with discrete up or down moves while progressing toward the finish line (Figure 13B). EEG recordings were conducted with NVX52 amplifier (Medical Computer Systems Ltd). We sampled 26 EEG channels at 500 Hz with the reference to A1-A2 (see the schematics of channels in Figure 13E). One paraplegic subject imagined leg movements during the motor-imagery state. The parameters of the spatial filter are shown in Figures 13C–F. Figure 13C shows a representative trace of the ICA component representing SMR. Figure 13D shows the spectrum of this signal, where two distinct peaks are present in the alpha and beta bands for the resting state data (blue). The spectrum shows a clear SMR desynchronization in the motor-imagery state (orange). Subject-specific frequency band (17–21 Hz), marked by vertical lines in Figure 13D, was selected. The spatial-temporal filter assured a good separation between the motor-imagery and resting states (Figure 13E). As depicted in Figure 13G, NFBLab based implementation of the foot motor-imagery BCI allowed the subject to achieve a very low command latency (< 200 ms) which resulted in nearly instantaneous gradual control of the game. The details of this experiment design are available on-line within the NFBLab GitHub resource^7^ Figure 13H shows the signal processing diagram implemented by the NFBLab in this example.

Moreover, this NFBLab based BCI implementation has been recently used to control the lower-limb exoskeleton in another paraplegic patient with complete leg paralysis. Several challenges had to be addressed in this exoskeleton paradigm, including eliminating mechanical, electrical and EMG artifacts. All these have been successfully handled by the NFBLab software.

## 9. Discussion

BCI and NFB paradigms are real-time EEG/ECoG/MEG paradigms that are relevant to both basic neuroscience and clinical applications. While the idea of recording neural activity and feeding it back to the subject dates back to the 1960s and 1970s, the BCI and NFB fields have experienced a renaissance during the last decade, with experts from multiple disciplines developing systems that extract and process neural information and convert it into commands to external devices or NFB. Research and development of the modern BCI and NFB systems require advanced software tools. Although the existing software for real-time EEG processing has been useful for many applications, we need a next-generation of tools to move forward and address problems related to improving specificity of the feedback signal which will lead to better efficacy of both NFB and BCI technologies. These new software tools should be sufficiently flexible to allow for development of new experimental protocols and their modifications. At the same time, the software should provide a core functionality, with essential EEG processing methods incorporated, such as removal of artifacts, detection and allocation of rhythmic components through spatio-temporal filtering, and evaluation of instantaneous power and phase of oscillatory EEG components. Furthemore, this software should be available to the broad cohort of researchers and should support a wide variety of encephalographic devices, including MEG equipment with unique combination of spatial and temporal resolution properties.

The proposed NFBLab platform offers a solution that meets all these requirements. It supports flexible configuration of the experimental protocol and contains a number of components for EEG data acquisition and processing. These components include functionality that allows to design signal processing chains having high spatial and temporal fidelity. The software inherently supports scripting which in turn allows for natural reproducibility of the paradigms and promotes sharing specific experimental designs by different researchers. Moreover, NFBLab is an open-source project that can incorporate new signal processing procedures and custom modules. The architecture of the software is modular and can be further extended by the community of users.

NFBLab uses the LSL interface that operates in two directions: multi-channel data acquisition, and streaming results of EEG data processing. This allows, on the one hand, for ensuring compatibility of NFBLab with the equipment of nearly all manufacturers, and on the other hand, implementing a variety of NFB stimuli and controlling an unrestricted variety of external devices.

NFBLab platform employs XML language to define signal processing pipelines, specify characteristics of the experimental blocks and determine the sequence of these blocks to be administered during the experiment. This allows for a fully automatic administration of the experiments with preset or randomized sequences of experimental blocks. Also, mock condition mode can be chosen blindly to the experimenter. This facilitates double-blind designs that must be used to establish the efficacy of NFB interventions. Additionally, XML scripts define the rules for combining different EEG-derived signals and for scaling the feedback based on the signal statistics. We believe that such scripting based approach facilitates reproducible neurofeedback and BCI research. XML scripts can be modified if needed both via NFBLab interface or via a standard text editor.

Although NFBLab offers a significant advantage over existing real-time EEG tools it still lacks certain features. In particular, a graphical programming interface once implemented would allow to create signal processing chains and experimental designs by dragging and dropping icons corresponding to specific signal processing operations and/or experimental protocols. LSL provides universal access to EEG data obtained by a broad variety amplifiers from various vendors. This universality comes at the cost of a delay introduced by the LSL protocol. Although not very significant in most of the applications, it may become a problem in certain cases. Therefore, we believe that implementing direct access to the DAC registers of EEG acquisition devices from some selected vendors. Then, in combination with the advanced signal processing algorithms, we will be able to explore the realm of zero-latency or negative latency feedback for the first time. Currently, only visual feedback is implemented. Adding auditory and tactile feedback options would further reduce efficient feedback delivery time because of the inherent properties of the corresponding sensory systems.

We have been successfully using this software in a wide range of our own projects related to real-time processing of electromyographic, electroencephalographic and electrocorticographic signals. Here we described in detail two implementations of EEG-based NFB and BCI paradigms, and mentioned the third one. The constantly growing functionality of the software goes beyond these examples. The compatibility of NFBLab platform with external hardware and software and its flexibility in adjusting experimental parameters make it useful for numerous research fields and clinical applications. In conclusion, NFBLab is a versatile software package that eases development of NFB systems and employs advanced algorithms to improve NFB efficiency. As such, it advances a variety of NFB and BCI applications, including NFB-based treatment of neurological conditions.

## 10. Software availability

The source code and installation instructions for NFBLab can be found at https://github.com/nikolaims/nfb. The manual is available at https://nfb-lab.readthedocs.io/en/latest/index.html. In order to facilitate learning how to use our software, we have developed a tutorial and included it in the documentation. In the provided example, the EEG device is emulated by an LSL stream created within the NFBLab and streaming the prerecorded data read from a file. After examining this example, a prospective user can follow the tutorial to get familiar with the basic concepts of NFBLab. The XML files implementing the NFB and BCI experiments described in section 8 are available at https://github.com/nikolaims/nfb/blob/master/tests/designs/alphanfbsettings.xml and https://github.com/nikolaims/nfb/master/tests/designs/bci2statessettings.xml.

## Author Contributions

NS developed the software and wrote the first version of the manuscript. KV tested the software and added application examples. SZ developed low-level routines for equipment support and maintained the equipment within application examples. ML provided general guidance, wrote and revised the paper. AO conceived the study, designed the software, developed the earlier version of the software, wrote and revised the paper.

### Conflict of Interest Statement

The authors declare that the research was conducted in the absence of any commercial or financial relationships that could be construed as a potential conflict of interest.

## Footnotes

^1^https://github.com/sccn/labstreaminglayer

^2^http://www.fieldtriptoolbox.org/development/realtime/buffer

^3^https://github.com/nikolaims/nfb

^4^http://openvibe.inria.fr/motor-imagery-bci/

^5^https://github.com/nikolaims/nfb/wiki/Experiment-file-structure

^6^https://github.com/nikolaims/nfb/blob/master/tests/designs/alpha_nfb_settings.xml

^7^https://github.com/nikolaims/nfb/master/tests/designs/bci_2states_settings.xml
